# Lipid nanoparticle-based mRNA vaccines in cancers: Current advances and future prospects

**DOI:** 10.3389/fimmu.2022.922301

**Published:** 2022-08-26

**Authors:** Tao Huang, Lushan Peng, Yingying Han, Dan Wang, Xiaoyun He, Junpu Wang, Chunlin Ou

**Affiliations:** ^1^ Department of Pathology, Xiangya Hospital, Central South University, Changsha, China; ^2^ Departments of Ultrasound Imaging, Xiangya Hospital, Central South University, Changsha, China; ^3^ Department of Pathology, School of Basic Medicine, Central South University, Changsha, China; ^4^ Key Laboratory of Hunan Province in Neurodegenerative Disorders, Xiangya Hospital, Central South University, Changsha, China; ^5^ National Clinical Research Center for Geriatric Disorders, Xiangya Hospital, Central South University, Changsha, China

**Keywords:** mRNA vaccine, nanocarrier, therapeutic target, delivery system, cancer therapy

## Abstract

Messenger RNA (mRNA) vaccines constitute an emerging therapeutic method with the advantages of high safety and efficiency as well as easy synthesis; thus, they have been widely used in various human diseases, especially in malignant cancers. However, the mRNA vaccine technology has some limitations, such as instability and low transitive efficiency *in vivo*, which greatly restrict its application. The development of nanotechnology in the biomedical field offers new strategies and prospects for the early diagnosis and treatment of human cancers. Recent studies have demonstrated that Lipid nanoparticle (LNP)-based mRNA vaccines can address the poor preservation and targeted inaccuracy of mRNA vaccines. As an emerging cancer therapy, mRNA vaccines potentially have broad future applications. Unlike other treatments, cancer mRNA vaccines provide specific, safe, and tolerable treatments. Preclinical studies have used personalized vaccines to demonstrate the anti-tumor effect of mRNA vaccines in the treatment of various solid tumors, including colorectal and lung cancer, using these in a new era of therapeutic cancer vaccines. In this review, we have summarized the latest applications and progress of LNP-based mRNA vaccines in cancers, and discussed the prospects and limitations of these fields, thereby providing novel strategies for the targeted therapy of cancers.

## Introduction

Messenger RNA (mRNA) vaccines represent a newly studied method of immunotherapy that works by introducing exogenous mRNA encoding antigens into the body and translating and synthesizing antigens in the cells, which finally result in an immune response (1, 2). mRNA was first discovered in 1961 (3), and after a decade, researchers discovered how to translate proteins from isolated mRNA in living cells (4). In the 1990s, scientists began to use mRNA expression vectors to inject mRNA into mouse somatic cells to express luciferase, chloramphenicol acetyltransferase and β-galactosidase (2). Studies in 1992 showed that diabetes insipidus occurred in mutant Brattleboro rats owing to their inability to express and secrete vasopressin. However, when copies of purified or synthesized mRNA from the hypothalamus of normal rats were injected into the hypothalamus, it was found that large cell neurons selectively ingested, retrogradely transported, and expressed vasopressin, and their diabetes insipidus could be temporarily reversed within a few hours after injection for up to 5 days (5). In recent years, mRNA vaccines have attracted increasing attention for the clinical treatment of various diseases and have become one of the most effective drugs. However, mRNA vaccines have not been widely used thus far, mainly because of their instability (6, 7). Determining a suitable method to improve the stability of mRNA vaccines is one of the focus areas in research in recent years. The ideal way to deliver mRNA is to use a material that can protect it from degradation and can induce the mRNA to be effectively absorbed by cells after injection (8).

**Figure 1:**
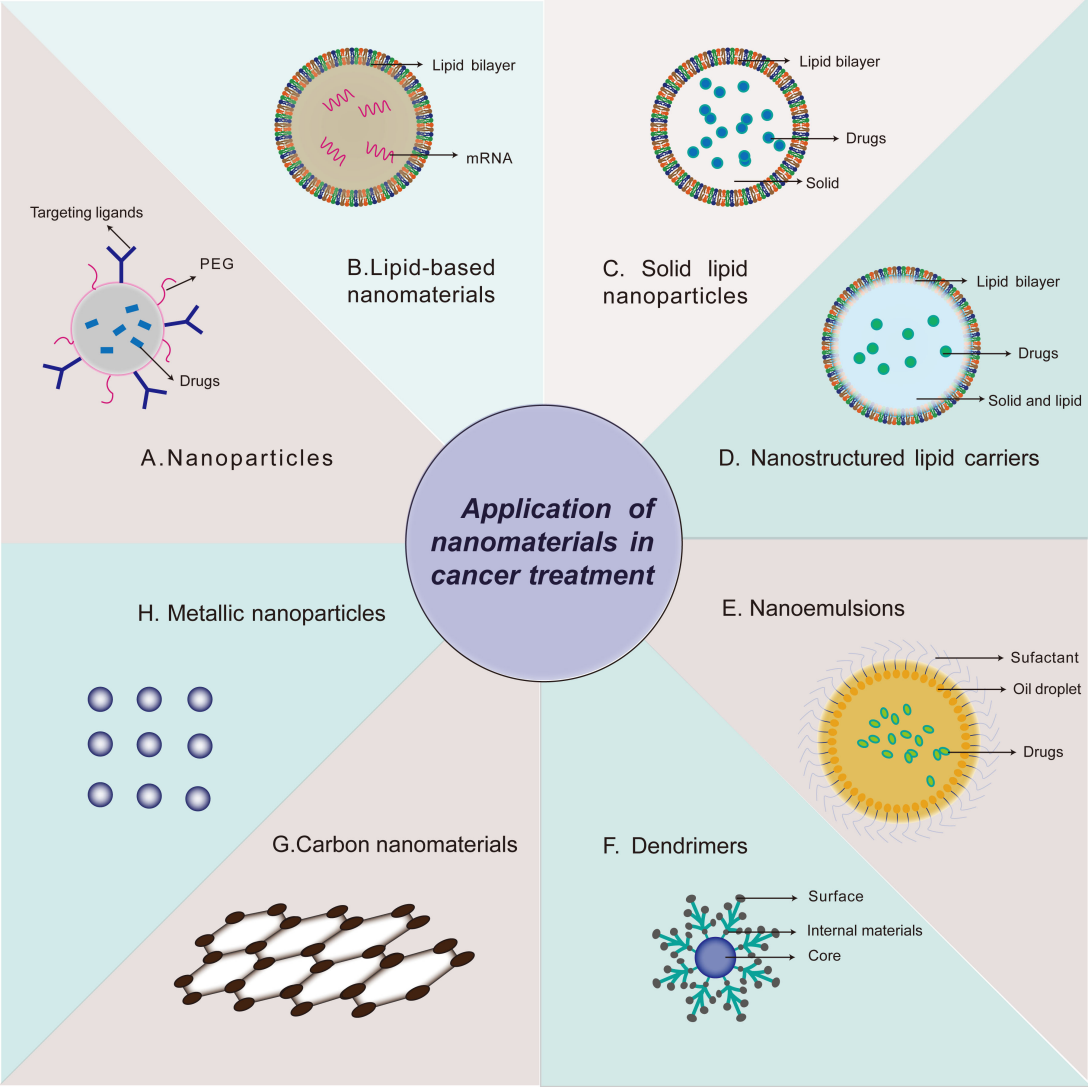
Strategies of nanomaterials applied in cancer treatment. Application of nanomaterials in cancer treatment, including Nanoparticles **(A)**, Lipid-based nanomaterials **(B)**, Solid lipid nanoparticles **(C)**, Nanostructured lipid carriers **(D)**, Nanoemulsions **(E)**, Dendrimers **(F)**, Carbon nanomaterials **(G)**, Metallic nanoparticles **(H)**.

According to the materials science standards, the term “nanotechnology” is used to describe the manufacture of new materials with sizes of 1 to 100 nm, but with further study, the definition of these new materials has also expanded (9). Nanomedicine is the application of nanomaterials in clinics, including diagnosis, detection, control, and treatment of diseases (10). Unlike bulk materials, nanomaterials are a new type of material with unique physical and chemical properties, such as ultra-small size, high reactivity, and large surface-area-to-volume ratio, and can be used to overcome the limitations of traditional therapeutic reagents (11). Nanomaterials are also similar to biomolecules in scale and can be designed as drugs with various functions (12). Nanotechnology has great potential for application in medicine. Delicate designs and modifications enable nano-drugs to maintain better specificity and bioavailability, low cytotoxicity to normal tissue, larger drug loading, longer half-life, and unique drug release patterns than traditional drug formats (13). The frequently-used strategies of nano-carrier in tumor therapy are shown in **Figure 1**. Studies have shown that nanocarrier-based mRNA vaccines are widely used in the treatment of diseases. At present, lipid nanoparticles (LNP) are the main nanocarriers for cancer treatment. Phospholipids are one of the main components of cell membranes. Many kinds of lipids such as C12-200, cKK-E12, 5A2-SC8, 306Oi10 and BAMEA-O16B have been identified that can be formulated into LNP (14). The design of LNP as a vector for delivering mRNA can overcome the following disadvantages of mRNA therapy: first, as a negatively charged macromolecule, mRNA has difficulty in crossing the cell membrane (15); second, the average intracellular half-life of mRNA is only about 7 h (16); third, a large amount of mRNA is trapped in the endosome after entering, and cannot translocate into the cytoplasm to perform the translation function (17).

Cancer is a severe disease that causes a global economic burden. Lung, colorectal, liver and breast cancers are the most common cancers (18). Various strategies can be used to deal with these serious diseases including surgery, radiotherapy, chemotherapy, immunotherapy, and microwave therapy, thereby greatly improving the therapeutic effect and quality of life of patients (19). However, for many patients, the above methods have obvious limitations owing to the serious side-effects and ineffectiveness of treatment. In recent years, nanotechnology has played an important role in vaccinology through the research on adjuvants and vaccine delivery systems (20–22). Moreover, some nanocarrier-based mRNA vaccines have been successfully applied to the treatment of coronavirus disease (COVID-19) (23–25), which has greatly enhanced the public’s confidence in the development of mRNA cancer vaccines. Further, some studies have reported the possibility of nanocarrier-based mRNA vaccines being used as cancer vaccines (26–32). Nanocarriers can address the instability of mRNA vaccines (33) and improve the targeting of drug therapy, which has good prospects through further research and the development of cancer nanocarrier-based mRNA vaccines.

## Structure and biological function of the mRNA vaccine

### Definition, structure and types of mRNA vaccines

The mRNA contains the genetic information of organisms, which can serve as a direct template for protein biosynthesis. It is a bridge that connects genetic information in the DNA with protein translation and expression, thereby playing an important role in regulating the activities of life. The mRNA vaccine, a subtype of the nucleic acid vaccine, can be divided into two categories, non-replicating mRNA and self-amplified RNA (saRNA). The traditional non-replicative mRNA consists of a 5′ cap, a 5′ - untranslated region (UTR), an open reading frame, a 3′- UTR and a poly (A) tail encoding a vaccine antigen. Structural elements are very important for the transcriptional efficiency and stability of mRNA. These elements are also modifiable sites, which can prolong the half-life of mRNA *in vivo* and limit unnecessary immune responses (34). Compared to conventional mRNA, saRNA is another kind of mRNA molecule with a different structure. saRNA can effectively produce a large amount of protein of interest by exploiting the innate nature of alphaviruses (17). The basic components of saRNA are the 5′ cap, 5′-UTR, sequence coding for nonstructural proteins (NSP), subgenomic promoter sequence, open reading frame, 3′-UTR, and a 3′ poly(A) tail (35). After saRNA is transfected into the cell, the NSP sequence is translated into the NSP polyprotein, which functions as the precursor of the replicase complex. This complex transcribes the original positive-sense RNA strand into a negative-sense RNA strand, which is then used as the template for subsequent replication (36).

Currently, there are four types of cancer vaccines: viral vector vaccines, tumor cell- and immune cell-based vaccines, peptide-based vaccines, and nucleic acid-based vaccines (37). Nucleic acid-based vaccines are promising platforms for cancer vaccines for many reasons. First, nucleic acid vaccines can simultaneously transmit multiple antigens covering a series of tumor-associated antigens (TAAs) or somatic tumor mutations, while stimulating cell-mediated and humoral immune responses, which significantly increase the possibility of overcoming vaccine drug resistance. Second, nucleic acid vaccines are unlike polypeptide vaccines, which can encode full-length tumor antigens, allowing antigen-presenting cells (APCs) to simultaneously present or cross-present multiple epitopes of class I and II patient-specific human leukocyte antigen (HLA). As a result, nucleic acid vaccines are not limited by HLA type and are more likely to stimulate a wider range of T-cell responses (38, 39).

## Mode of action and function of mRNA vaccines

**Figure 2:**
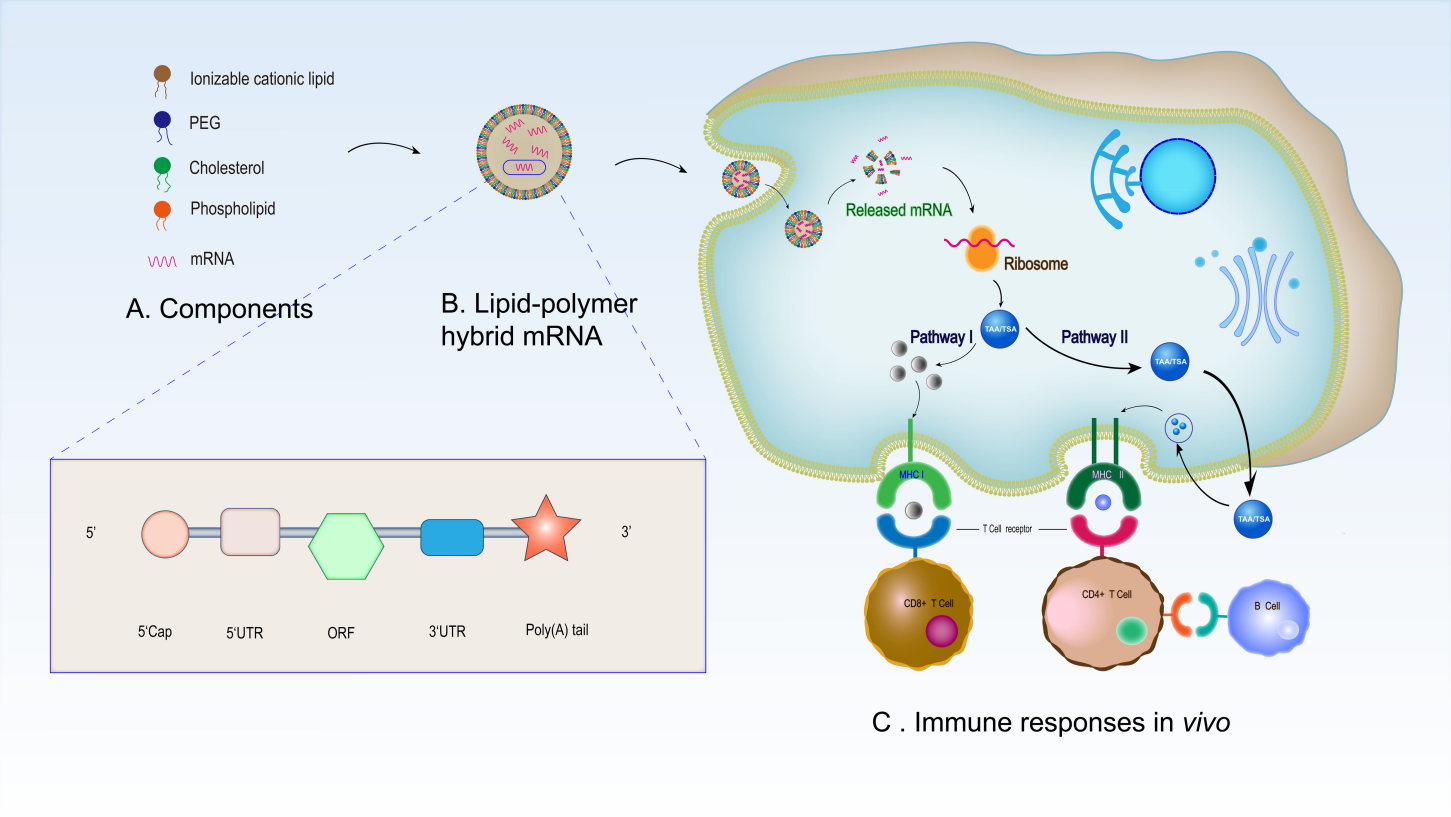
Synthesis and mechanisms of LNP-based mRNA vaccine in immunotherapy. **(A)** Synthetic raw materials of LNP -based mRNA vaccine. **(B)** Morphology of the assembled mRNA. The mRNA structure in the liposome is composed of a 5’cap structure, 5’- and 3’- UTR, a 3’ poly **(A)** tail and an open reading frame. **(C)** Immune responses of LNP-based mRNA vaccine *in vivo*. After entering the human body, the LNP-based mRNA vaccine enters antigen-presenting cells through endocytosis before releasing the mRNA in the cells. The released mRNA translates the target antigen in the ribosome. Subsequently, endogenous antigens are degraded into polypeptides and are presented by MHC I and activate CD8+ T cells (pathway I). In addition, secreted antigens can be taken up by cells, degraded inside endosomes, and presented on the cell surface to CD4+ T cells by MHC II. Finally, CD4+ T cells stimulate B cells to promote B cell maturation and then B cells produce specific antibodies to play an anti-tumor role (pathway II).

Some mRNA vaccines for non-cancerous diseases have been studied in clinical trials, especially in COVID-19 treatment (**Table 1**). Several preclinical and clinical studies have explored mRNA vaccines for anticancer use, either by adoptive transfer on dendritic cells (DCs) *in vitro* or by direct injection (47, 48). The basic principle of mRNA as a cancer vaccine is that it can deliver the target transcript encoding one or more tumor-specific antigens (TSAs) or TAAs to the cytoplasm of the host cell (especially in APCs), and then express it as the target antigen (49). Through a major histocompatibility complex, the expressed TSAs and TAAs can be presented on the cellular surface of APCs, thereby activating the anti-tumor immunity (50). When inoculated into host cells, mRNA can be translated into the corresponding antigens, imitating humoral and cellular immunity similar to a viral infection (51). mRNA vaccines enhance the antiviral and antitumor activity of the host by increasing the antigenic activity of T cells (52, 53). Most studies on T-cell immunity induced by antigen-encoded mRNA use autologous DCs, which are loaded with mRNA *in vitro* and reintroduced into patients. Although clinical trials have proved the safety and feasibility of this method, the *in vitro* operation of DCs requires complex personalized vaccination procedures, which seriously hinders its application in patients. Therefore, there is an urgent demand for new vaccination methods that can directly target DCs *in vivo* (1). We have described the process of using mRNA vaccines for drug treatment (**Figure 2**) (13).

**Table 1:** Clinical trials of mRNA vaccines in infectious diseases.

Disease type/Virus type	mRNA vaccine	Phase	Ref.
SARS-CoV-2	BNT162b1	I	(40)
	BNT162b2	III	(41)
	mRNA-1273	III	(42)
HIV-1	iHIVARNA	II	(43)
Rabies	CV7201	I	(44)
	CV7202	I	(45)
Respiratory Syncytial Virus	mRNA-1777 (V171)	I	(46)

Many drugs use nanomaterials as carriers. Compared to other treatment with small molecules, DNA, oligonucleotides, viral systems and proteins (including antibodies), messenger RNA therapy has many advantages (54). In contrast to oligonucleotides and most small molecular drug targets, mRNA vaccines can simultaneously mediate stimulation and inhibition patterns, as well as express or replace defective proteins, thus expanding the range of their potential indications. Compared to DNA vaccines and lentiviral vector, mRNA vaccine only needs to enter the cytoplasmic ribosomal translation mechanism and not the nucleus; thus, there is no risk of genome integration (55–59). Furthermore, after mRNA vaccination, antigen expression is transient, thus avoiding T cell depletion caused by persistent antigen exposure (60). Compared to protein vaccines, mRNA vaccines have the advantages of simple synthesis, fast purification and low cost (61).

### Common methods for improving the stability of mRNA vaccines

Because of the degradation of RNases *in vivo* and the innate immunogenicity of mRNA vaccines, mRNA is easily degraded before APCs are present. Under the influence of these factors, improving the stability of mRNA can ensure an expression effect. The common ways of improving the stability of mRNA translation are as follows (**Table 2**). For example, using synthetic cap analogues that by binding to the eukaryotic translation initiation factor 4e (eIF4E) stabilize mRNA and increase protein translation (73, 74); regulatory elements in the 5’- and 3’- UTR and poly (A) tail also can stabilize mRNA and increase protein translation (75, 76); nucleoside modification reduces the activation of innate immunity and enhances translation (77); and sequence and/or codon optimization increases translation (78). Although changing the codon composition or introducing modified nucleosides can positively regulate protein expression, these may affect the secondary structure of mRNA (79), kinetics and accuracy of protein folding and translation (80), and the expression of T cell epitopes in alternative reading frames (81). Collectively, these factors may affect the degree and specificity of the immune response.

**Table 2:** Strategies for efficient translation of mRNA.

Strategy	Introduction	Function	Example	Ref.
Synthetic cap analogues and capping enzymes	Composed of 7-methylguanosine (m7G) linked by a 5′− 5′-triphosphate bridge to the first transcribed nucleotide.	Binding of the 5′ cap to eukaryotic translation initiation factor 4E(eIF4E) is crucial for efficient translation,	M7GpppN, M7GpppG	(62–64)
UTR	The 5′ and 3′ UTR of eukaryotic mRNAs play crucial roles in the post-transcriptional regulation of gene expression	Play crucial roles in the post-transcriptional regulation of gene expression through the modulation of nucleocytoplasmic mRNA transport, translation efficiency, subcellular localization and message stability	Human β-globin 3′-UTR	(65–67)
Poly(A) tail	The 3′poly(A) tail, in association with poly(A) binding proteins (PABP), plays a critical role in eukaryotic mRNA metabolism	Regulates the stability and translational efficiency of mRNA in synergy with the 5′ cap, the internal ribosomal entry site and various other determinants	—	(68, 69)
Sequence and/or codon optimization	Sequence and/or codon optimality is a major determinant of mRNA stability	Sequence and/or codon optimality is a major determinant of mRNA stability	—	(70)
Modified nucleosides	Some mRNA modifications have been shown to affect normal development	Decrease innate immune activation and increase translation	m6A, m5A, m1A, pseudouridine	(71, 72)

### Structural and functional features of LNP-based mRNA vaccines

The study of mRNA vaccines based on nanomaterial delivery systems can overcome the limitations of *in vivo* delivery, such as insufficient protein expression in cells, insufficient antigen load, and maturation of APCs (82). Nanotechnology has made an important contribution to the development of effective vaccine adjuvants and delivery systems. LNP-based mRNA vaccines can protect the encapsulated mRNA from the host environment and release it continuously to induce a lasting immunostimulatory effect. Nanocarriers, including viral and non-viral carriers, have also been explored as potential tools in delivering vaccines because they are expected to elicit a wide range of immune responses in addition to cell-mediated immunity (83). Nanomaterial delivery systems mainly include nanoemulsion-based adjuvants, lipid nanocarriers, and adjuvants targeting pattern recognition receptors (83). The use of nanomaterials as drug carriers is effective in the treatment of many diseases such as pulmonary infectious diseases and psoriasis (84–86).

Currently, the main nanomaterials used to deliver mRNA vaccines are lipid nanoparticles (LNPs). LNPs constitute four components, including ionizable cationic lipids, lipid-linked polyethylene glycol, cholesterol, and naturally occurring phospholipids. The synthesis of LNPs is a crucial process, which directly affect the size and encapsulation efficiency of the LNPs. The crucial factor in the synthesis of fine and uniform LNPs is the rapid mixing of the excess water and ethanol-lipid phase. Using the staggered herringbone structure, microfluidic mixing is a selectable synthesis method, for the diverse-scale synthesis of LNPs (15). Once the mRNA vaccines enter the targeted cells, the process of their forming complexes with cationic lipids released is necessary for nucleic acid delivery. Through neutralizing the charge of their cationic lipid carriers, the cell’s anionic lipids can help LNPs release nucleic acids, thereby disrupting the electrostatic interactions between the lipid carriers and nucleic acids (87, 88).

The extracellular half-life of mRNA can be prolonged by encapsulating mRNA into liposome nanoparticles to prevent its degradation by ubiquitous RNA enzymes (89–92). Nanocarrier administration can also improve the half-life of biological agents by preventing the premature release and degradation of drugs and evading clearance from the kidneys and liver (93). Thus, a nanomaterial-based delivery system can maintain the extracellular stability of the mRNA vaccine.

## Application of LNP-based mRNA vaccines in cancer therapy

**Figure 3:**
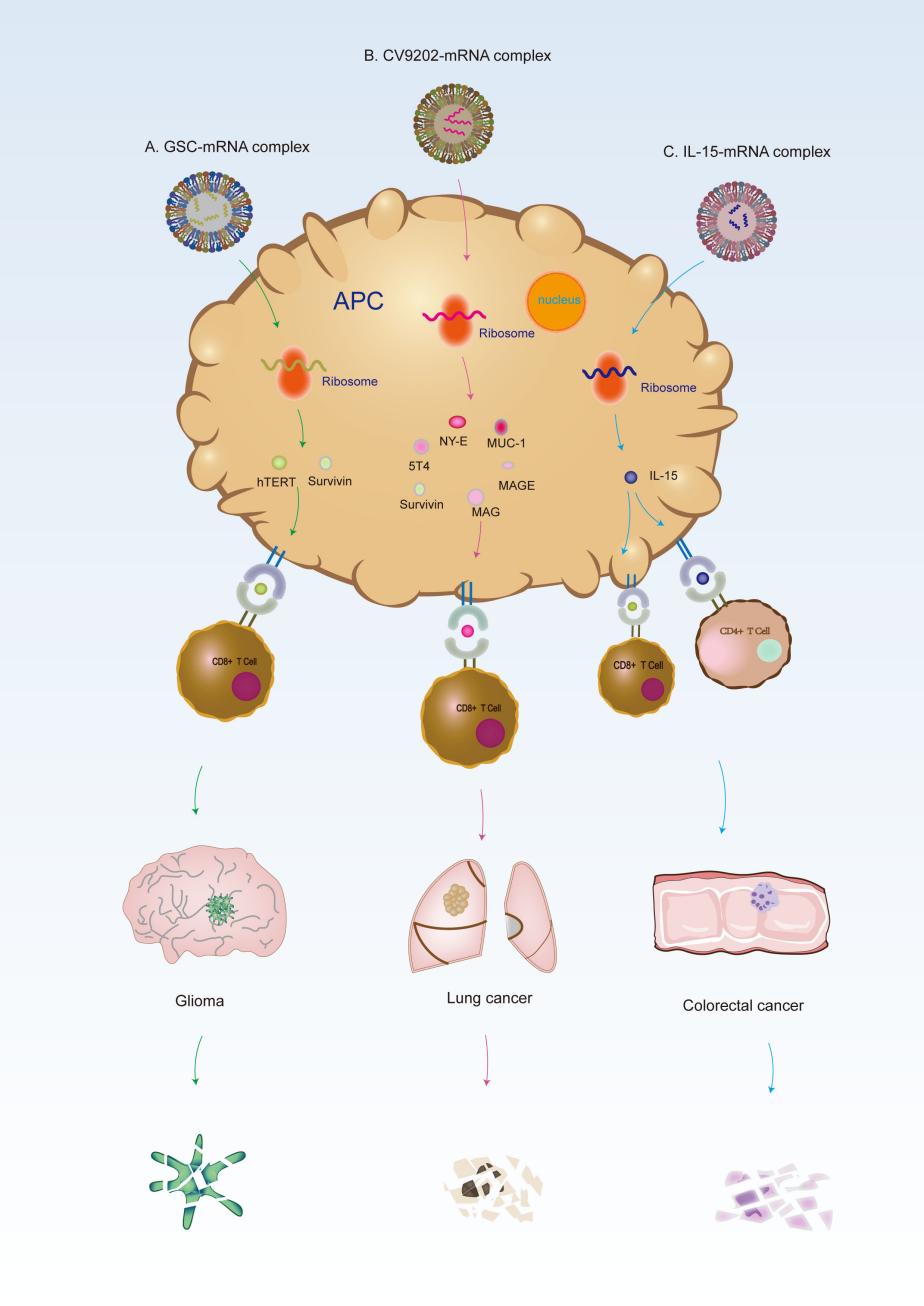
Mechanism of mRNA vaccines against lung cancer, CRC and glioma. **(A)** Glioma stem cells (GSC): Autologous dendritic cells transfected with autologous GSC-mRNA are utilized to induce an immune response against the GSC of patients. GSC-mRNA can translate hTERT and survivin to induce the immune response. **(B)** Lung cancer: CV9202 is a cancer immunotherapy with sequence-optimized mRNAs encoding different cancer antigens in free and complexed form with the CLPP; this facilitates antigen expression and activation of the immune system, essentially conferring self-adjuvant activity and subsequently inducing an adaptive cellular and humoral immune response. **(C)** CRC: After the CLPP/mRNA complex enters the body, CLPP can deliver the IL-15-mRNA to antigen-presenting cells (APCs), and then APCs will translate IL-15 protein and release it, causing cellular immune response and promoting the cell death of CRC.

LNP-based mRNA vaccines are more selective than other treatments. The use of nanomaterials for drug delivery can reduce adverse reactions by preventing the non-specific absorption of therapeutic agents in healthy tissues (94). Therefore, this is more suitable for patients who cannot tolerate traditional treatment because of their poor physical condition. Targeted therapy has also been proven to be an effective method for tumor therapy (95, 96). Because of their benign biocompatibility and low toxicity, endogenous carriers show great potential for the delivery of therapeutic nanoparticles (97, 98). An efficient way to deliver drugs to the lesion is to wrap anticancer agents in nanocarriers (99–101). The main advantage of the LNP-based strategy compared to naked mRNA is that it can deliver many drugs specifically by changing the pharmacokinetic properties of drugs, which increases drug intake in tumor tissues, improves the anti-tumor effect, and reduces non-specific toxicity (102, 103). Currently, many immunotherapies based on mRNA have been applied in clinical trials, and some confirmative results have shown the efficacy results of solid tumor treatments (54). Cancer therapies involve multi-disciplinary participation (104), and the vaccine is in the developing stage. LNP-based mRNA vaccines have obvious advantages, but they have not been widely used in clinical practice. LNP-based vaccines that are currently in the preclinical phase or have been used in clinical trials have been introduced below. **Figure 3** shows the anti-cancer mechanism of LNP-based mRNA vaccines in lung cancer, colorectal cancer (CRC), and glioma.

### LNP-based mRNA vaccines in lung cancer

Lung cancer is the most commonly diagnosed cancer, and the leading cause of cancer-related deaths (105). Non-small-cell lung cancer (NSCLC) is the most common type of lung cancer, with approximately 40% patients having stage IV metastatic disease when diagnosed (106). According to the type and stage of malignant tumors, the treatment of lung cancer usually requires a combination of chemotherapy, surgery, or radiotherapy. However, because of the inadequacy of early diagnosis, the cancer in most patients with lung cancers is detected at an advanced stage, with distant metastasis or local tumor invasion, and are not suitable for surgical treatment. With the progression of the disease, the survival time of patients in stage IV lung cancer gradually decreases to 4 months. Therefore, it is crucial to intervene at an early stage. Systemic cytotoxic therapy has become the main treatment for advanced NSCLC, but the benefits of chemotherapy have reached a plateau and new forms of treatment are needed (107). Systemic chemotherapy is the mainstream treatment for advanced lung cancer; it aims to prolong the survival time of patients and improve their quality of live (108). The standard first-line chemotherapy regimen for lung cancer includes platinum-based drugs (e.g. carboplatin and cisplatin) (109). However, platinum-based chemotherapy causes dose-limiting side effects, including intestinal damage, anemia, nephrotoxicity and cardiotoxicity, and peripheral neuropathy, with other symptoms such as nausea, restlessness, and fatigue (110). Moreover, the survival rate of traditional chemotherapy combinations has stabilized, and the median survival rate of patients with molecular changes targeted by new drugs is only approximately 2–3 years (111).

Currently, only a few preclinical studies on LNP-based mRNA vaccines are available for lung cancer. In a clinical Phase Ib study, Papachristofilou et al. (112) demonstrated that BI1361849, an immunotherapeutic that included the five antigens encoded by CV9202, combined with local radiation treatment with or without pemetrexed was well tolerated with few side effects, and could induce a targeted immune response in patients with stage IV NSCLC. After vaccination, functional CD4^+^ and/or CD8^+^ T cells in 40% of patients increased at least twice as much as before treatment compared to the baseline. This treatment provided evidence to support that BI1361849 (CV9202) combined with immune checkpoint inhibitors benefitted the patients with NSCLC. Similarly, in a clincal phase I/IIa study, Sebastian et al. (113) concluded that the antigen-specific immunotherapy (CV9201) was well tolerated and had enhanced the immune response in patients with stage IIIB/IV NSCLC. Encouragingly, the efficacy and safety of mRNA vaccines (BI 1361849) combined with a checkpoint inhibitor, anti-CTLA-4(tremlimumab) and anti-PD-L1(duvalumab) in the treatment of NSCLC have been evaluated in an ongoing phase I/II study (NCT03164772) (114). These studies suggested the importance of mRNA-based immunotherapy combined with immune checkpoint inhibitors in the treatment of NSCLC.

### LNP-based mRNA vaccines in colorectal cancer

In 2018, more than 1.8 million new CRC cases and 881,000 deaths were estimated to have occurred (105). Currently, most stage I or II CRC patients are treated surgically, and the standard surgical procedure for CRC is total mesenterectomy (115, 116). Surgery with chemotherapy is considered the standard treatment for patients with stage III CRC, whereas systemic chemotherapy or a combination of targeted biologics is often preferred for patients with metastatic CRC (117).

Regarding the close relationship between the incidence of CRC and heterogeneity (118), precision therapy has been used for treating CRC in recent years (19, 119–121). LNP -based mRNA vaccines display unique advantages for the therapy of CRC. Lei et al. (122) established a liposome/protamine system (CLPP) nano-delivery system to provide IL-15-encoded mRNA for colon cancer gene therapy. The results indicated that the IVTIL-15mRNA could be effectively and safely introduced into CT26 CRC cells through the prepared CLPP system. Local administration as well as systemic administration of the CLPP/mIL-15 complex showed significant anticancer effects in a C26 CRC cell model of subcutaneous metastasis, abdominal metastasis and lung metastasis through various mechanisms. Local or systemic administration of CLPP/mIL-15 complex had an obvious inhibitory effect on a variety of C26 CRC mouse models, with inhibition rates of 70%, 55%, and 69% in the CT26 abdominal metastasis model, subcutaneous metastasis model and in the lung metastasis model, respectively. They are all highly effective and safe. After vaccination, the activity of lymphocytes was significantly stimulated, and the magnitude of CD8^+^T cells increased significantly. These data proved that the CLPP/mIL-15 complex had high therapeutic potential in the immune gene therapy of CRC, indicating that CLPP is ideal for mRNA delivery, and the CLPP/mIL-15 complex is promising for cancer immune gene therapy.

### LNP-based mRNA vaccines in glioma tumor

Central nervous system tumors and peripheral nervous system tumors together constitute neurotumors. The formation of these tumors is mainly caused by improper nerve repair after a certain degree of nerve injury (123). Although only a small proportion of the neurotumor cases are the central nervous system tumors (105), their treatment is challenging. It is challenging to reach the tumor site surgically owing to the deep location of the central nervous system. Therefore, as the current treatment still has many limitations, exploring the regulatory mechanism and searching for biomarkers for glioma tumors is urgently need (124).

mRNA can be used to treat genetic diseases or repair tissues as they express certain functional proteins; thus, they can also be used for immunotherapy by expressing antigens, antibodies or receptors. Although these therapies are not sufficiently mature, they have broad research prospects. Vik-Mo et al. (125) successfully isolated brain tumor biopsy tissues and prepared a single-cell suspension. Autologous glioma stem cells (GSCs) were amplified into tumor spheres *in vitro*, and GSC-mRNA was amplified and transfected into monocyte-derived autologous DCs. Autologous DCs transfected with autologous GSC-mRNA were used to induce an immune response to the patient’s GSCs. Compared to somatic neural progenitor cells, GSCs had enhanced telomerase activity and highly expression of survivin, an inhibitor of apoptosis protein. Seven patients were treated with DC vaccine targeting GSCs in solid tumors, resulting in a 2.9 times increase in the progression-free survival of patients with glioma. In all seven patients, they found specific-induced lymphocyte proliferation upon stimulation with tumorsphere lysate, hTERT, or survivin peptides.

### LNP-based mRNA vaccines in other tumors

In 2010, Provenge (Sipuleucel-T), an immune cell-based vaccine approved by the United States Food and Drug Administration, served as the first therapeutic cancer vaccine for patients with hormone-resistant prostate cancer (126). Huang et al. (127) found that CD247, FCGR1A and TRRAP were potential antigens of mRNA vaccines against cholangiocarcinoma, especially for patients with the immune subtypes 2 (IS2) tumors, providing a theoretical foundation for the development of anti-cholangiocarcinoma mRNA vaccines and identifying suitable vaccination targets. Huang et al. (128) also reported that ADAM9, MET, TPX2, EFNB2, WNT7A, and TMOD3 were effective antigens of the anti-pancreatic cancer mRNA vaccine, and that patients with two immune subtypes IS4 and IS5 tumors were suitable for vaccination. The anti-cancer vaccines CV9103 and CV9104 can also be used for the treatment of prostate cancer (129).

### LNP-based mRNA vaccines in targeted therapy of cancer

**Figure 4:**
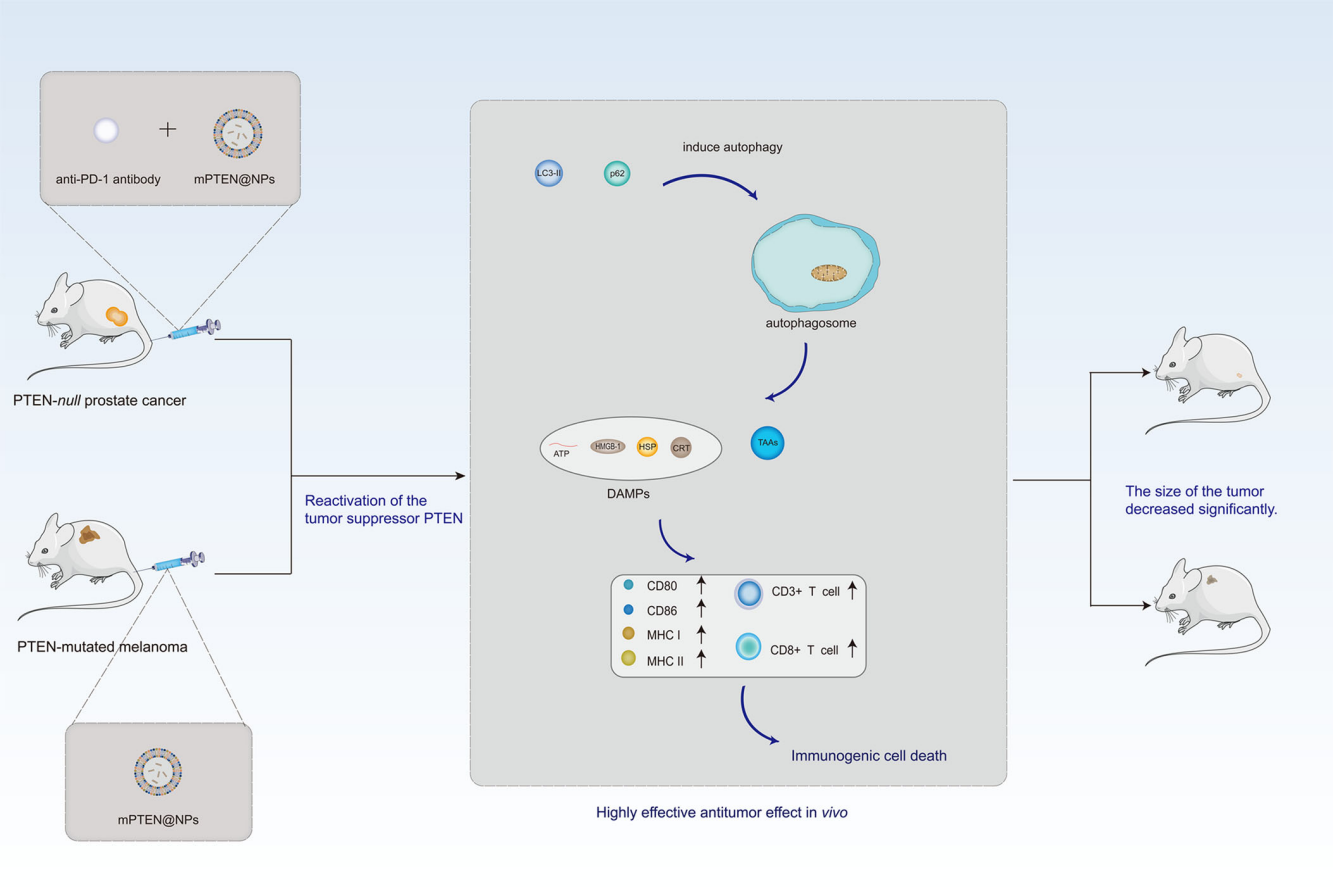
Antitumor function of PTEN can be reactivated by PTEN mRNA nanoparticles (mPTEN@NPs) in preclinical models. In the *Pten*-mutated melanoma and *Pten*-null prostate cancer mouse model, the combination of PTEN mRNA nanoparticles (mPTEN@NPs) can reverse the inhibition of antitumor immune responses by increasing the infiltration of CD8+T and CD3+T cells, as well as combine with anti-programmed death-1 antibody enhancing the therapeutic efficacy.

Although the prospect of LNP-based mRNA vaccines as a cancer therapy is optimistic, currently, its application is mainly in the translational therapy stage. Translational medicine is usually defined as “the transfer of new understandings of disease mechanisms gained in the laboratory into the development of new methods for diagnosis, prevention, and therapy” (130). As shown in **Figure 4**, in the *Pten*-mutated melanoma and *Pten*-null prostate cancer mouse model, the combination of PTEN mRNA nanoparticles (mPTEN@NPs) can reverse the inhibition of antitumor immune responses by increasing the infiltration of CD8+T and CD3+T cells, and can combine with anti-programmed death-1 (anti-PD-1) antibody enhancing the therapeutic efficacy (131). Moreover, Zhang et al. (132) used paclitaxel aminolipid (PAL)-derived nanoparticles to integrate p53-mRNA and chemotherapy drug. In contrast to clinical drugs, PAL-p53-mRNA nanoparticles had higher paclitaxel loading. In addition, these nanoparticles showed the synergistic cytotoxicity of paclitaxel and P53-mRNA in cultured TNBC cells. They displayed the anti-tumor effect of PAL-p53-mRNA nanoparticles *in vivo* in a TNBC mouse model. In **Table 3**, we summarized the current application of LNP-based mRNA vaccines in conversion therapy, aiming to provide new methods and ideas for clinical treatment in the future. The applications of LNP-based mRNA vaccines in cancer transformation therapy are limited, while transformation therapy is an extremely important stage before the clinical application of drugs, which is the premise and basis of drugs entering clinical trials. Taken together, we hope that more drug therapeutic targets can be confirmed in transformation trials.

**Table 3:** LNP-based mRNA vaccines in translational medicine.

Tumor types	Nanoparticles	mRNA	Targets	Model	Ref.
Prostate cancer, melanoma	mPEG-PLGA, G0-C14	PTEN	CD8+T cell ↑	Mice	(131)
Prostate cancer, breast cancer	mPEG-PLGA, G0-C14	PTEN	Suppress the PI3K–AKT pathway	Mice	(133)
Triple-Negative breast cancer	Lipid/calcium/phosphate (LCP)	MUC1	Elicit the killing efficiency of antigen-specific CD8+cells	Mice	(134)
Melanoma	Graphene oxide (GO), polyethylenimine (PEI)	OVA	CD8+T cell ↑	Mice	(135)
Triple-negative breast Cancer	Paclitaxel amino lipid (PAL), camptothecin amino lipid (CAL)	P53	—	Mice	(132)

## Conclusion and prospect

LNP-based mRNA vaccines are an excellent example of combining materials′ science with medicine, enhancing communication between different disciplines, and integrating the different innovative approaches. Antagonism between humans and cancer is perennial. As an emerging cancer therapy, mRNA vaccines have broad future applications because unlike other treatments, cancer mRNA vaccines provide specific, safe, and well-tolerated treatments. Preclinical studies have used personalized vaccines to evaluate the anti-tumor effect of mRNA vaccines in the therapy of several solid tumors, including lung cancer and CRC. To further improve the capacity of mRNA anti-cancer vaccines, many clinical trials are underway to evaluate the efficacy and safety of mRNA vaccines in combination with checkpoint inhibitor therapy or cytokine therapy. As a new method of cancer therapy, the significant advantages of LNP-based mRNA vaccines are reflected in the following aspects: (1) the significant advantage of mRNA vaccines compared to other treatment lies in the efficacy, safety, and productive efficiency of mRNA vaccines; (2) with the rapid development of nanotechnology, LNP-based mRNA vaccines are likely to greatly improve the stability of mRNA vaccines, prevent mRNA from decomposing prematurely *in vivo*, increase the half-life of drugs, and reduce the dose and frequency of drugs; (3) with an in-depth understanding of the mechanism of cancer, the targeting of LNP-based mRNA vaccines will be further improved. mRNA vaccines can treat diseases from the genetic level. Compared with other therapies, LNP-based mRNA vaccines are more scientific, have few side effects and will not cause other adverse effects in patients, which is a good embodiment of the benefits of precision medicine in cancer treatment.

However, the clinical application of LNP-based mRNA vaccines is limited by several factors. First is the high diversity of tumor antigens. Cancer can be caused by multiple genes, which is a challenge in the development of mRNA vaccines. Second limitation is determining the proper treatment targets. The correct treatment target requires accurate determination of the location and related information about the genes that cause the disease. Although the Human Genome Project has been completed, the locations of many disease-associated genes are still unknown, which renders mRNA vaccines lack effective therapeutic targets for diseases of unknown etiology, resulting in rare types of cancers that could potentially be treated with mRNA vaccines. Third limitation is the availability of suitable nanocarriers. Although liposomes are currently the most widely used nanocarriers of mRNA vaccines, they still have a certain toxicity and are not absolutely safe for the human body. It is difficult to search for a safer and more efficient nanocarrier because a suitable nanocarrier requires identifying materials that not only combine perfectly with the drugs, but also avoid rejection by human cells and need to be of low toxicity. This requires further development of materials′ science. Fourth limitation is the high capital needs. The research and development of mRNA vaccines are still in its infancy, with uncertain efficacy in animals, and requiring more clinical trials in humans to prove that they are efficacious; hence, this process requires considerable financing.

In conclusion, LNP-based mRNA vaccines have shown potential in the treatment and prevention of cancers because of their evident advantages, and are expected to be a promising approach in many cancer therapies. Moreover, it is reasonable to believe that LNP-based mRNA vaccines could play an important role in disease diagnosis and prognosis in the near future.

## Author contributions

CO, JW and XH conceived manuscript. TH collected relevant references, drafted manuscript and finished the figures. All authors offered crucial content revision and language polishing. CO, JW and XH completed the final manuscript. All authors contributed to the article and approved the submitted version.

## Funding

This work was supported by the National Natural Science Foundation of China (No. 81602167 and 81903032), the China Postdoctoral Science Foundation (No. 2020M672520), the Outstanding Youth Foundation of Hunan Provincial Natural Science Foundation of China (No. 2022JJ20098), the Hunan Provincial Natural Science Foundation of China (No. 2021JJ31100 and 2021JJ41013), the Science and Technology Program Foundation of Changsha City (No. kq2004085) and the Youth Fund of Xiangya Hospital (No. 2018Q011).

## Conflict of interest

The authors declare that the research was conducted in the absence of any commercial or financial relationships that could be construed as a potential conflict of interest.

## Publisher’s note

All claims expressed in this article are solely those of the authors and do not necessarily represent those of their affiliated organizations, or those of the publisher, the editors and the reviewers. Any product that may be evaluated in this article, or claim that may be made by its manufacturer, is not guaranteed or endorsed by the publisher.

## Abbreviations

CRC, colorectal cancer; COVID-19, Corona Virus Disease 2019; UTR, untranslated region; TAAs: tumor-associated antigens; APCs: antigen-presenting cells; HLA, human leukocyte antigen; DCs, dendritic cells; TSAs, tumor-specific antigens; MHC, major histocompatibility complex; eIF4E, eukaryotic translation initiation factor 4E; m7G, 7-methylguanosine; LNPs, lipid nanoparticles; NSCLC, Non-small-cell lung cancer; CLPP, a liposome/protamine system; IL-15, Interleukin 15; GSCs, glioma stem cells; mPTEN@NPs, PTEN mRNA nanoparticles; PD-1, programmed death-1; PAL, paclitaxel aminolipid; TNBC, Triple-negative breast cancer.
